# A case of IgA nephropathy treated with a combination of telitacicept and half-dose glucocorticoids

**DOI:** 10.1515/biol-2025-1158

**Published:** 2025-08-28

**Authors:** Suojian Zhang, Di Yin, Qin Xu, Caixia Zhao, Jianmei Sha, Zhenguo Qiao, Juan Cao

**Affiliations:** Department of Nephrology, Taixing People’s Hospital, Taizhou, 225400, Jiangsu, China; Department of Gastroenterology, Suzhou Ninth People’s Hospital, Suzhou Ninth Hospital Affiliated to Soochow University, Suzhou, 215200, Jiangsu, China

**Keywords:** IgAN nephropathy, telitacicept, half-dose glucocorticoids

## Abstract

Immunoglobulin A nephropathy (IgAN) is the most common primary glomerular disease in China; there is an urgent need to identify more effective treatments for IgAN. A 34-year-old woman presented with proteinuria of >2 years’ duration. She was diagnosed with IgA nephropathy and was treated with a combination of telitacicept and half-dose glucocorticoids. At the time of writing, the patient’s urinary protein concentration and renal function were normal. A combination of telitacicept and half-dose glucocorticoids is an effective way to treat IgAN.

## 1 Introduction

Immunoglobulin A nephropathy (IgAN) is the most common primary glomerular disease in China. According to the results of a study performed at the General Hospital of Eastern Theater Command, IgAN accounts for 52.66% of cases of primary nephropathy [1]. Furthermore, the study showed an increase in the incidence of IgAN over the previous decade [1]. Approximately 30–40% of patients with IgAN develop renal failure within 20–30 years of developing the condition [2]. Therefore, there is an urgent need to identify more effective treatments for IgAN. A recent phase II clinical study of the treatment of IgAN with telitacicept showed that 240 mg reduced proteinuria by 49% and 160 mg reduced proteinuria by 25% vs placebo [3]. Here, we report the treatment of a patient with IgAN using methylprednisolone 24 mg/day in combination with telitacicept 160 mg weekly, which achieved good results. This treatment was approved by the ethics committee of our hospital.

## 2 Case presentation

A 34-year-old woman presented with proteinuria of >2 years’ duration. In 2021, the patient was found to have protein in her urine, but no edema, and no action was taken. Subsequently, her urine protein continued to be positive, and her urine albumin/creatinine ratio (ACR) was 1,506 mg/g in July 2023. She had no previous history of significant ill health. Laboratory testing revealed urine protein 2+, urine red blood cell count 66/μL, 24-h urine protein 2,941 mg, hemoglobin 141 g/L, serum C-reactive protein 1.49 mg/L, serum albumin 41.5 g/L, serum creatinine 90 μmol/L, IgA 3.43 g/L, IgG 0.872 g/L, and IgM 3.47 g/L. Her liver enzymes, serum lipid concentrations, blood glucose, and serum uric acid were normal. She was negative for autoantibodies, anti-neutrophil cytoplasmic antibodies, antibodies to the phospholipase A2 receptor (PLA_2_R), HBV, HCV, and HIV. B-mode ultrasonography showed that her right and left kidneys were 93 mm × 39 mm × 11 mm and 101 mm × 46 mm × 14 mm in size, respectively. Chest computed tomography and electrocardiography showed no abnormalities.

**Figure 1:**
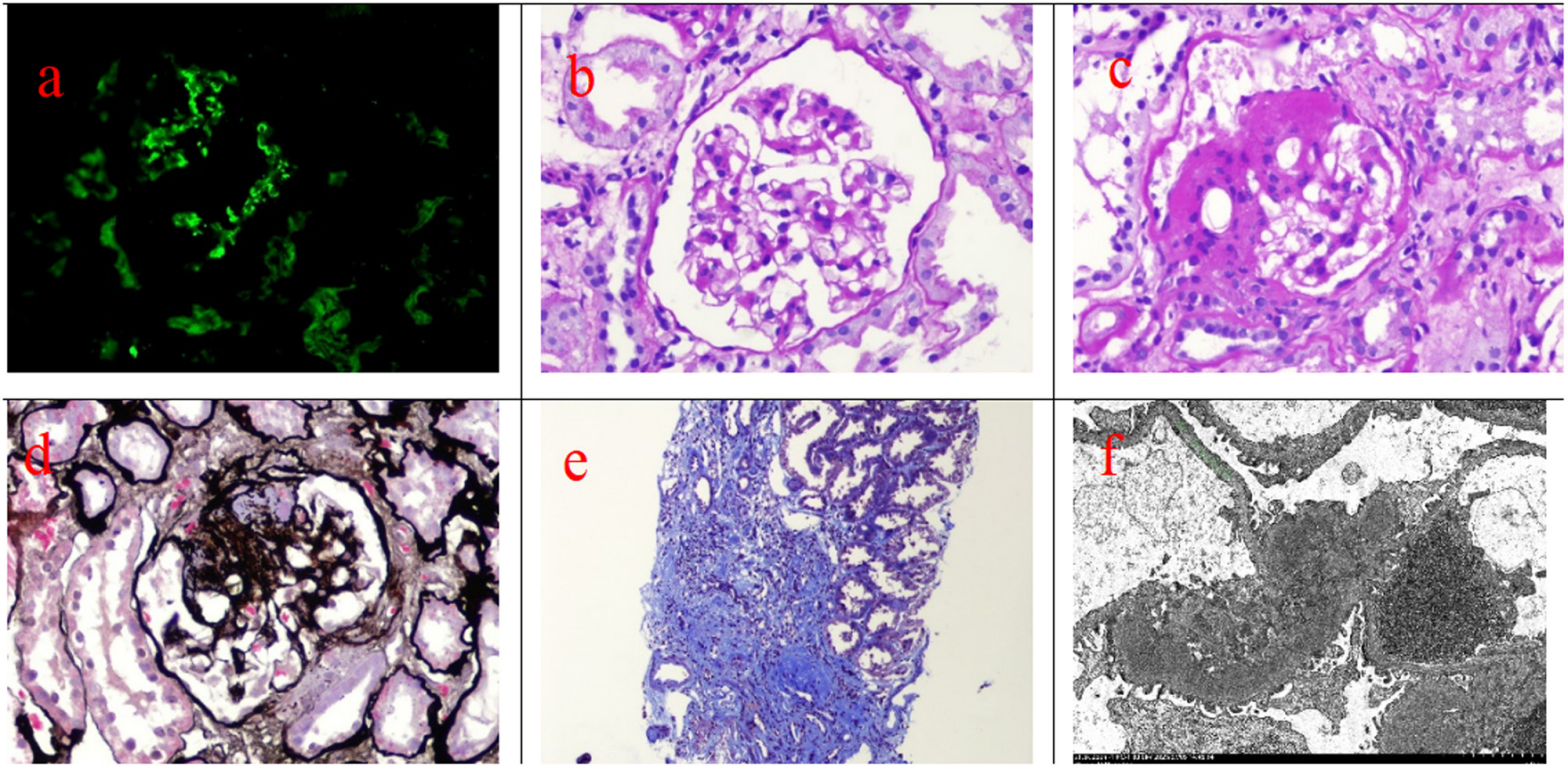
Renal pathological results of this patient. (a) Immunofluorescence staining of biopsied renal tissue showed the positive IgA deposition in the mesangial area (×400). (b) PAS staining exhibited mild mesangial proliferation in the glomerulus (×400). (c) PAS staining showed afferent and efferent arteriolar hyalinosis (×400). (d) PASM staining showed the glomeruli with segmental glomerulosclerosis (×400). (e) Masson trichrome staining showed obvious tubulointerstitial fibrosis (blue stained, ×100). (f) Transmission electron microscope observed electron-dense mesangial deposits (×15,00).

Renal biopsy was performed on July 15, 2023. Immunofluorescence revealed that the five glomeruli were IgG−, IgA+++, IgM++, C3+−, C1q−, and FRA−. These antibodies were present in the mesangial area as granular masses (Figure 1a). Light microscopy showed that there were 15 glomeruli in the specimen, 5 of which showed glomerulosclerosis and 1 of which showed segmental sclerosis with balloon adhesion, mild diffuse proliferation of glomerular mesangial cells and matrix, and focal segmental lesions, but no endothelial cell proliferation. There were also severe tubulointerstitial lesions, multiple foci of atrophy of the renal tubules, granular or vacuolar degeneration of tubular epithelial cells, flat or necrotic exfoliation or regeneration of segmental renal tubular epithelial cells, occasional exposure of the renal tubular basement membrane, and large numbers of protein casts and red blood cells, as well as a lot of cell debris in the lumen. Multifocal mononuclear cell infiltration with fibrous connective tissue hyperplasia was apparent in 55% of the renal interstitium (Figure 1b–e). Transmission electron microscope observed electron-dense mesangial deposits (Figure 1f). The clinical and fluorescence and light microscopic findings were consistent with a diagnosis of focal proliferative sclerosing IgAN (Hass grade III; Oxford classification: M1E0S1T2C0).

**Figure 2:**
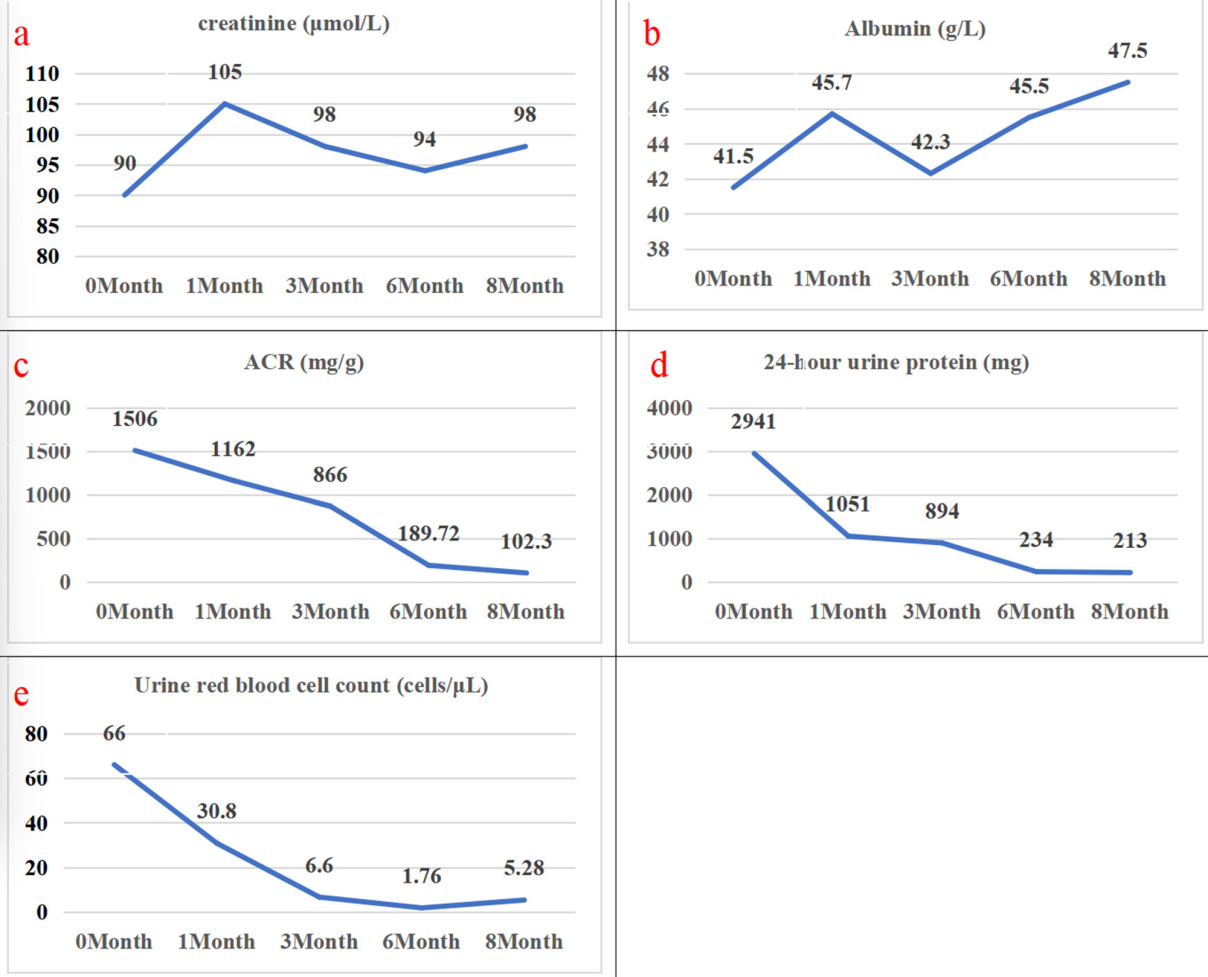
Serum creatinine, albumin, urine albumin creatinine ratio (ACR), 24-hour urine protein and urine red blood cell counts. (a) Serum creatinine remained relatively stable. (b) Serum albumin showed an increasing trend. (c) ACR showed a decreasing trend. (d) 24-hour urine protein showed a decreasing trend. (e) Urine red blood cells showed a decreasing trend.

**Figure 3:**
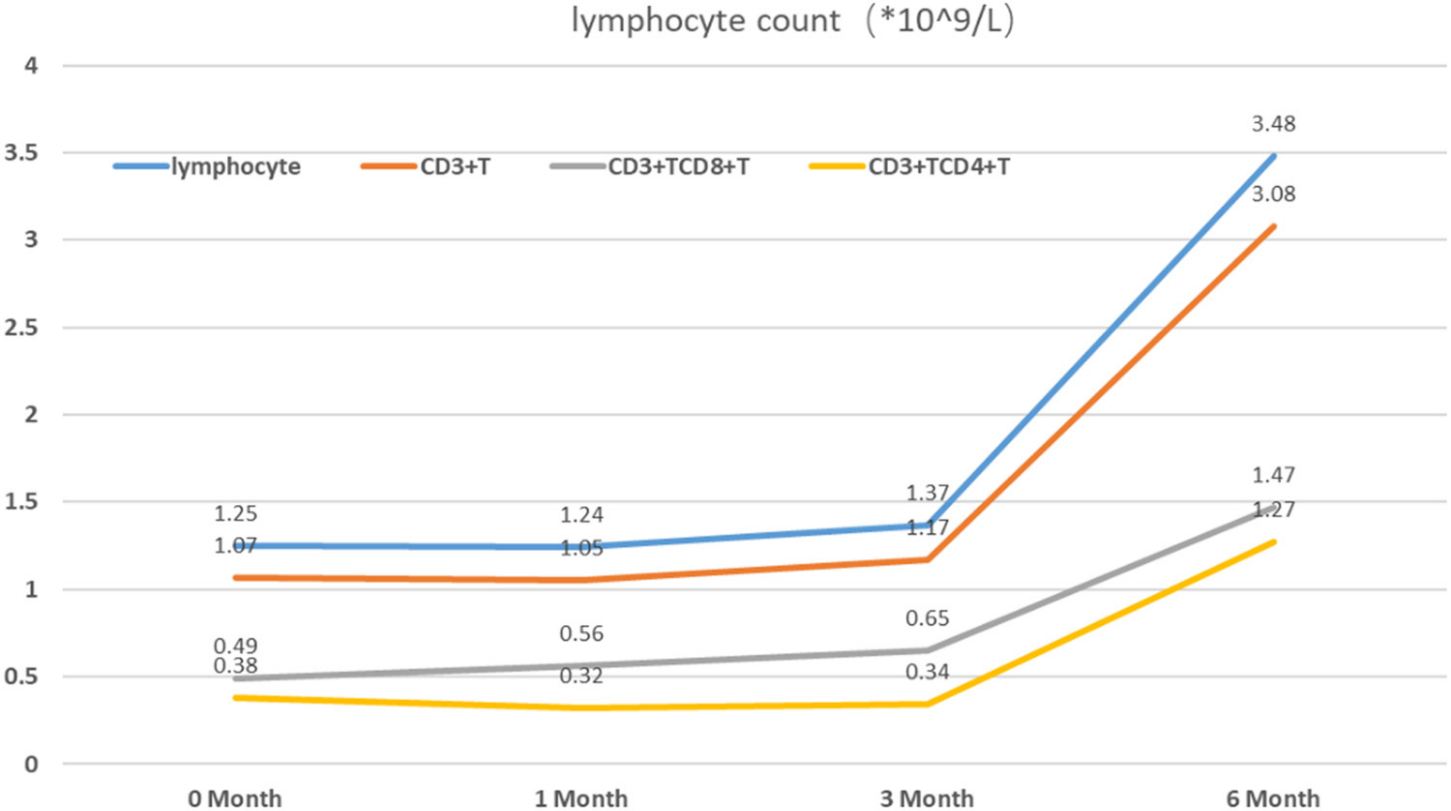
Lymphocyte counts during treatment.


Treatment of the patient commenced on July 19, 2023, in the form of a subcutaneous injection of telitacicept 160 mg weekly, and oral methylprednisolone 20 mg/day and valsartan 80 mg/day. By January 3, 2020, a 24-week course of treatment had been completed, and the regimen was changed to telitacicept 160 mg every 15 days and methylprednisolone 4 mg/day for maintenance. At the time of writing, the patient’s urinary protein concentration and renal function were normal. Her serum creatinine, plasma albumin, 24-h urinary protein, urinary ACR, and urine red blood cell counts are shown in Figure 2. Lymphocyte counts are shown in Figure 3.

**Informed consent:** Informed consent has been obtained from all individuals included in this study.
**Ethical approval:** The research related to human use has been complied with all the relevant national regulations and institutional policies and in accordance with the tenets of the Helsinki Declaration and has been approved by the authors’ institutional review board or equivalent committee.

## 3 Discussion

IgAN is a common primary glomerular disease with various clinical manifestations. Persistent massive proteinuria, hypertension, pathological changes, and renal dysfunction are risk factors for a poor prognosis [4]. At the time of admission, the patient had moderate proteinuria (urine protein 2,941 mg/day) and a relatively high serum creatinine concentration. Although her blood pressure was normal at the time, her renal pathology was severe, involving global sclerosis in 5 of 15 glomeruli, and segmental sclerosis with balloon adhesion in another, accompanied by multifocal mononuclear cell infiltration and fibrous connective tissue hyperplasia covering 55% of the renal interstitium. Without treatment, the prognosis of this patient would have been relatively poor.

At present, the pathogenesis of IgAN is not fully characterized, but the “four-hit theory” is generally accepted. The first of these is the production of galactose-deficient IgA1 (Gd-IgA1) and the activation of B cells to produce IgG or IgA antibodies against Gd-IgA1. This antibody binds to Gd-IgA1 to form a circulating immune complex, which is eventually deposited in the mesangium, where it activates the complement cascade and the inflammatory response, leading to kidney injury [5]. Therefore, the targeting of Gd-IgA1 and antibody production represents a promising method of treatment.

In the TESTING study, patients diagnosed with IgA nephropathy were administered glucocorticoids at a dosage of 0.6–0.8 mg/kg per day. Notably, compared to the placebo group, those in the glucocorticoid group exhibited significantly higher rates of both complete and partial remission, underscoring the efficacy of this treatment approach. However, during an average follow-up period of 19.7 months (ranging from 9.2 to 30.1 months) within the glucocorticoids group, a concerning 11% of patients experienced serious adverse events, particularly infections [6]. In our daily clinical practice, we frequently encounter individuals with IgA nephropathy whose proteinuria levels fail to decrease consistently. When treated with standard doses of glucocorticoids in conjunction with immunosuppressants, these patients are more susceptible to recurrent and even severe infections. This susceptibility complicates their treatment regimen and, unfortunately, accelerates the progression of their disease. A randomized controlled study showed that rituximab is an effective means of eliminating B cells but fails to reduce the Gd-IgA1 and Gd-IgA1 antibody titers and does not reduce the proteinuria of patients with IgAN, indicating that it is not an effective treatment for IgAN [7]. Budesonide has shown promising therapeutic effects in IgA nephropathy by significantly lowering serum levels of Gd-IgA1 and inhibiting the production of pathogenic antibodies [8]. However, this drug was not yet available when she was diagnosed with IgA nephropathy. B lymphocyte stimulator (BLyS) and A proliferation-inducing ligand (APRIL) are two key mediators of B-cell maturation and activation [9], and telitacicept inhibits the further development and maturation of immature B cells by blocking BLyS and inhibiting the differentiation of mature B cells into plasma cells by blocking APRIL, and affecting the secretion of autoreactive plasma cell autoantibodies, and inhibiting Gd-IgA1 and anti-Gd-IgA1 antibodies, thereby reducing immune complex deposition in the kidneys, breaking the immune activation cycle in chronic inflammation, and mitigating continuous kidney damage, thereby controlling disease activity [10,11]. The phase II clinical study conducted by Lv et al. showed that telitacicept 240 and 160 mg reduce proteinuria by 49 and 25%, respectively, vs placebo [3]. For the present patient, we administered a combination of a half-dose of methylprednisolone and telitacicept therapy to reduce the side effects associated with a full dose of the steroid and the required dose of telitacicept, thereby reducing the economical burden on the patient. The patient was relatively young, and after some discussion, she agreed to be treated using telitacicept, and was administered a half dose of methylprednisolone (24 mg/day) in combination with telitacicept 160 mg/day, in addition to valsartan 80 mg/day, which had a good therapeutic effect. After 6 months of treatment, the patient had achieved complete remission, and her telitacicept regimen was changed to 160 mg every 2 weeks. After 8 months, she remained in complete remission, her plasma albumin concentration had gradually increased, and her serum creatinine concentration was stable. During the entire follow-up period, the patient’s lymphocyte count was stable, with no significant decline, and she did not experience any infections. In the future, we plan to study the efficacy of telitacicept in combination with a half-dose of glucocorticoid for the treatment of IgAN in additional patients.
